# Cefiderocol Targeted Treatment for Multidrug-Resistant Gram-Negative Infections: An Observational Cohort Study

**DOI:** 10.3390/antibiotics15040416

**Published:** 2026-04-20

**Authors:** Lourdes García-Carnero, Gabriela Abelenda-Alonso, Marc Santos-Puig, Ariadna Padullés, Clara Ribera, Alberto Lamiel, Rosa Costa-Primo, Manuel González de Aledo, Rosa Granada, Víctor Daniel Gumucio, Eva Santafosta, Marc Gilabert, Alejandro Blanco-Arévalo, Mireia Puig-Asensio, Evelyn Shaw, Jordi Carratalà, Carlota Gudiol

**Affiliations:** 1 Infectious Diseases Department, Bellvitge University Hospital, 08907 L’Hospitalet de Llobregat, Spain; lourdesgarcia.05@gmail.com (L.G.-C.); ablancoar@bellvitgehospital.cat (A.B.-A.); mpuiga@bellvitgehospital.cat (M.P.-A.); evelyn.shaw@bellvitgehospital.cat (E.S.); jcarratala@bellvitgehospital.cat (J.C.);; 2 Bellvitge Biomedical Research Institute (IDIBELL), 08908 L’Hospitalet de Llobregat, Spain; apadulles@bellvitgehospital.cat; 3 Research Network for Infectious Diseases (CIBERINFEC), Instituto de Salud Carlos III, 28029 Madrid, Spain; 4 Pharmacy Department, Bellvitge University Hospital, 08907 L’Hospitalet de Llobregat, Spain; marcsantos@bellvitgehospital.cat (M.S.-P.); cribera@bellvitgehospital.cat (C.R.); alamiel@bellvitgehospital.cat (A.L.); 5 Microbiology Department, Bellvitge University Hospital, 08907 L’Hospitalet de Llobregat, Spain; rmcosta@bellvitgehospital.cat (R.C.-P.); manuelgonzalez@bellvitgehospital.cat (M.G.d.A.); 6 Department of Intensive Care, Bellvitge University Hospital, 08907 L’Hospitalet de Llobregat, Spain; rgranada@bellvitgehospital.cat (R.G.); vgumucio@bellvitgehospital.cat (V.D.G.); esantafosta@bellvitgehospital.cat (E.S.); marcgilabert@bellvitgehospital.cat (M.G.); 7 Institut Català d’Oncologia (ICO), Hospital Duran i Reynals, 08908 L’Hospitalet de Llobregat, Spain

**Keywords:** cefiderocol, multidrug-resistant Gram-negative bacteria, carbapenem-resistant Enterobacterales, *Stenotrophomonas maltophilia*, *Pseudomonas aeruginosa*

## Abstract

**Background/Objectives**: Infections caused by multidrug-resistant Gram-negative bacteria (MDR-GNB) represent a major therapeutic challenge, particularly in hospitalized and critically ill patients with limited treatment options. Cefiderocol, a novel siderophore cephalosporin, has demonstrated activity against a broad range of resistant Gram-negative pathogens. We aimed to evaluate the effectiveness and safety of cefiderocol for the treatment of MDR-GNB infection. **Methods**: We conducted a retrospective observational study including all adult patients who received ≥72 h of cefiderocol between November 2020 and October 2024 at a Spanish tertiary-care hospital. The primary outcome was clinical success, defined as survival and absence of clinical recurrence 30 days after cefiderocol initiation. Secondary outcomes included 30- and 90-day mortality, clinical and microbiological recurrence, emergence of resistance, and adverse events. **Results**: Eighty patients were included (median age 64 years [IQR 56–72]; 81.3% male). Respiratory (26.2%) and abdominal (22.5%) infections were the most common, and 20% presented with bacteremia. At infection onset, 26.2% had septic shock and 45% required intensive care unit admission. The three most frequently isolated pathogen was *Pseudomonas aeruginosa* (33.9%), followed by Enterobacterales (33%) and *Stenotrophomonas maltophilia* (30.1%). Clinical success was achieved in 67.5% of patients. Thirty and 90-day mortality rates were 27.5% and 36.5%, respectively. Recurrence within 90 days occurred in 5% of cases. Emergence of resistance was detected in one *Klebsiella pneumoniae* ST147 isolate, and serious adverse events occurred in 5% of patients. **Conclusions**: In a cohort including a substantial proportion of critically ill patients, cefiderocol was associated with favorable clinical outcomes and an acceptable safety profile. These findings suggest that cefiderocol may represent a useful therapeutic option for severe MDR-GNB infections in patients with limited treatment alternatives.

## 1. Introduction

The global rise in multidrug-resistant (MDR) Gram-negative bacterial infections represents a major public health concern. These pathogens are associated with reduced susceptibility to conventional antibiotics, which may lead to prolonged hospitalizations, increased healthcare costs, and higher mortality rates [1,2]. In Europe, OXA-48-like carbapenemases are the most commonly reported among Enterobacterales, whereas KPC has been more frequently described in the United States. In addition, metallo-β-lactamases (MBLs), including NDM and VIM, have been increasingly reported in both regions [3,4,5,6]. In Spain, carbapenemase epidemiology shows marked heterogeneity, with OXA-48 predominating in Enterobacterales and VIM-type enzymes representing the main MBL mechanism, particularly in *Pseudomonas aeruginosa*; historically, treatment has relied mainly on colistin- or aminoglycoside-based regimens, often with limitations [7].

As available treatments decline, the need for new antimicrobials targeting MDR Gram-negative bacilli (MDR-GNB) has become urgent. Cefiderocol, a novel siderophore cephalosporin, utilizes bacterial iron-uptake pathways to enhance intracellular entry, and retains activity against strains producing a broad range of β-lactamases, including MBL [8]. Although randomized controlled trials have demonstrated its efficacy and safety [9,10,11], real-world data are crucial to better characterize its performance in complex clinical scenarios, informing optimal use, dosing adjustments, microbiological outcomes, and safety profiles. Reports of clinical practice cefiderocol use are increasing but remain limited. Therefore, we aimed to assess the effectiveness of cefiderocol for treating MDR-GNB infections in a tertiary-care hospital in Spain.

## 2. Results

### 2.1. Patient Characteristics

A total of 80 patients received cefiderocol between November 2020 and October 2024. The median age was 64 years (interquartile range [IQR] 56–72), and 81.3% were male (Table 1). Immunosuppression was present in 25% of patients, and the median Charlson Comorbidity Index was 3 (IQR 2–7). The most common reasons for hospital admission were abdominal sepsis (17.5%), urinary sepsis (13.7%), and acute pancreatitis (11.2%).

### 2.2. Clinical Characteristics

As shown in Table 2, the most frequent source of infection was respiratory (26.2%), followed by abdominal (22.5%). Bacteremia was identified in 16 patients (20%), and 26.2% presented with septic shock. The median Pitt bacteremia score was 5 (IQR 3–7). Regarding critical care needs, 45% required ICU admission, 32.5% required invasive mechanical ventilation, 20% underwent continuous renal replacement therapy (CRRT), and 6.3% received extracorporeal membrane oxygenation (ECMO).

### 2.3. Microbiological Data

**Figure 1:**
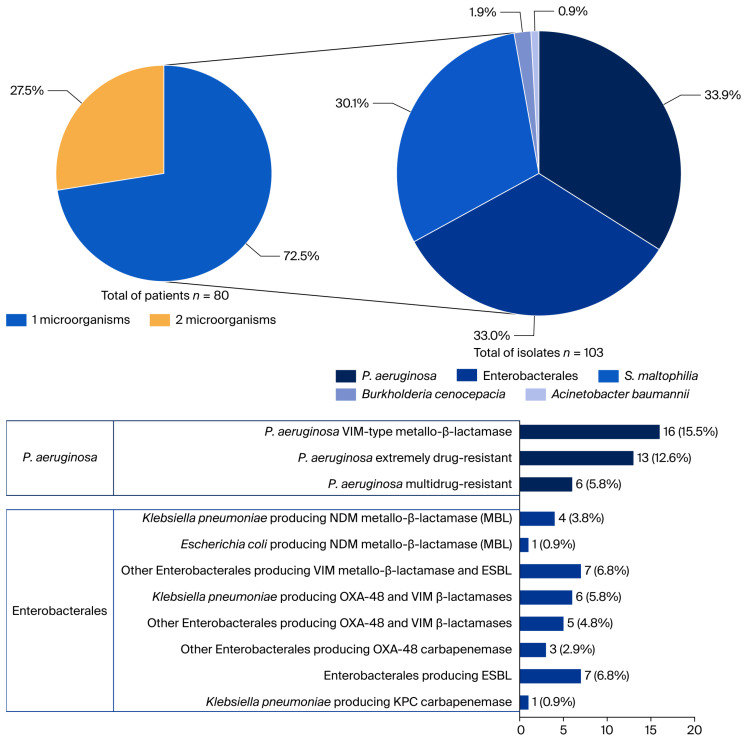
Microbiological distribution of isolates. (**Left panel**): distribution of monomicrobial (58/80, 72.5%) and polymicrobial (22/80, 27.5%) infections among patients included in the study. (**Right panel and lower panel**): distribution of causative microorganisms based on the total number of isolates identified in the study. A total of 103 isolates were identified 58 from monomicrobial infections and 45 from polymicrobial infections. VIM: Verona integron-encoded metallo-β-lactamase (a carbapenem-hydrolyzing enzyme); NDM: New Delhi metallo-β-lactamase (carbapenemase); MBL: metallo-β-lactamase (β-lactamase requiring zinc; often confers carbapenem resistance); ESBL: extended-spectrum β-lactamase (hydrolyzes extended-spectrum cephalosporins); OXA-48: oxacillinase-48 (class D β-lactamase; can confer carbapenem resistance); KPC: *Klebsiella pneumoniae* carbapenemase (class A carbapenemase).

A total of 58 patients presented an MDR-GNB monomicrobial infection (72.5%), while 22 patients (27.5%) presented a polymicrobial infection. As seen in Figure 1, a total of 103 isolates were identified, 58 from monomicrobial infections and 45 from polymicrobial infections. The three most frequently isolated pathogens were *Pseudomonas aeruginosa* (33.9%), Enterobacterales (33%) and *Stenotrophomonas maltophilia* (30.1%). Polymicrobial infections most frequently involved combinations of *S. maltophilia* with Enterobacterales, particularly ESBL-producing *K. pneumoniae*, harboring OXA-48 and/or VIM (*n* = 3), and ESBL-producing *E. coli* (*n* = 3). Combinations of VIM-producing *P. aeruginosa* with Enterobacterales were also common, including *K. pneumoniae* ESBL-NDM (*n* = 3), *E. cloacae* ESBL-VIM (*n* = 2), *E. coli* ESBL-NDM (*n* = 1), and *K. oxytoca* ESBL-VIM (*n* = 1). Of note, ESBL-producing Enterobacterales were identified exclusively in polymicrobial infections involving additional multidrug-resistant pathogens. A detailed breakdown of microorganisms and their associated resistance mechanisms in monomicrobial and polymicrobial infections is provided in Supplementary Tables S1 and S2. Cefiderocol MICs were available for 50 isolates (Table S3): MIC50 values were 0.25 mg/L for Enterobacterales (range 0.016–1 mg/L), 0.047 mg/L for *S. maltophilia* (range 0.016–0.25 mg/L), and 0.38 mg/L for *P. aeruginosa* (range 0.023–2 mg/L). Of note, resistance emergence during therapy was rare, documented in only one case involving a *K. pneumoniae* strain with a complex resistance profile, including New Delhi metallo-β-lactamase (NDM), OXA-48 carbapenemase, and extended-spectrum β-lactamase (ESBL) production. This isolate belonged to the high-risk ST147 clone, and the patient received monotherapy with cefiderocol.

### 2.4. Use of Cefiderocol

Cefiderocol was used as targeted therapy in all cases, with a median treatment duration of 9.5 days (IQR 5.25–20.5). Overall, 21.2% of patients received combination therapy, most commonly with aerosolized colistin (13.5%), followed by intravenous aminoglycosides (5.0%) and intravenous colistin (2.5%). Before starting cefiderocol, 20% had received empirical carbapenem therapy, and 13.5% had received ceftazidime–avibactam with or without aztreonam. In all such cases, switching to cefiderocol was prompted by coinfection with *S. maltophilia*. Details on aminoglycoside and colistin combination therapy, including infection sources and routes of administration, are provided in Supplementary Table S4.

The total median cefiderocol dose was 6 g per day (IQR 4.5–6). All ICU patients received treatment by continuous infusion, as well as two of the eight patients with osteoarticular infection. Of the ICU patients, only one patient received an increased dose of 8 g by continuous infusion over 24 h due to augmented renal clearance (>130 mL/min). The remaining patients received treatment at doses adjusted for their renal function by extended infusion over 3 h. None received an initial loading dose.

### 2.5. Primary and Secondary Outcomes

As presented in Table 3, clinical success was achieved in 67.5% of patients. The 30-day and 90-day all-cause mortality rates were 27.5% and 36.5%, respectively. Clinical success rates by focus of infection are provided in Table 3. Clinical recurrence at 30 and 90 days was observed in 4 (5%) and 2 (2.4%) patients, respectively. Microbiological recurrence at 90 days occurred in only one patient colonized with an NDM–OXA-48–ESBL-producing *K. pneumoniae* ST147 strain. No significant differences in clinical or microbiological outcomes were observed between patients treated with cefiderocol monotherapy and those receiving combination therapy. Serious adverse events leading to discontinuation occurred in 4 patients (5%), including rash, dizziness, myoclonus, and thrombocytopenia. Clinical outcomes stratified by infection type (monomicrobial vs. polymicrobial) and by causative microorganisms are summarized in Table S5. Factors potentially associated with treatment failure are summarized in Supplementary Table S6.

## 3. Discussion

In this retrospective cohort study, we assessed the effectiveness and safety of cefiderocol for the treatment of MDR-GNB infections in a tertiary-care hospital in Spain. We observed a clinical success rate of 67.5% and a 30-day all-cause mortality rate of 27.5%, findings that are consistent with previous randomized controlled trials [10,11,12], subsequent predefined subanalyses [13], and observational setting studies [14,15,16,17,18,19,20,21]. Before cefiderocol initiation, 13.5% of patients had received ceftazidime/avibactam with or without aztreonam. In episodes involving *S. maltophilia*, cefiderocol was selected based on microbiological and practical considerations, particularly in polymicrobial infections or when aztreonam availability was limited, as previously described in observational studies [15,17].

The clinical success rate in our cohort aligns with results from the CREDIBLE-CR trial (54–70%) and the APEKS-NP trial, in which cefiderocol demonstrated non-inferiority to high-dose meropenem for Gram-negative nosocomial pneumonia. Notably, our cohort included patients with substantial disease severity, reflected by high rates of septic shock, ICU admission, and mechanical ventilation—characteristics often underrepresented in randomized trials. Despite this, our outcomes were comparable to those reported in several observational studies. In this context, the high proportion of polymicrobial infections observed in our cohort likely contributed to the complexity of clinical management, as cefiderocol was primarily directed against the MDR Gram-negative pathogen, while additional co-pathogens often required concomitant therapy. Clinical outcomes in these cases therefore depended not only on cefiderocol activity, but also on adequate companion antimicrobial coverage, source control, and infection severity. In our cohort, treatment failure appeared to be more frequent among patients with severe clinical presentation, including septic shock and ICU admission, as well as in those with *P. aeruginosa* infections (particularly VIM-producing and XDR strains) and polymicrobial infections, especially of intra-abdominal origin. These findings are consistent with previous studies highlighting disease severity, difficult-to-treat *P. aeruginosa*, and complex infection sources as key determinants of poor outcomes in MDR Gram-negative infections [18,19,21]. Notably, although a higher Charlson Comorbidity Index has been associated with increased mortality in MDR Gram-negative infections, its role as a predictor of treatment response is less clear. In our cohort, similar Charlson scores between groups suggest that outcomes were more likely driven by disease severity and infection-related factors rather than baseline comorbidity.

Some multicenter observational studies have supported cefiderocol as a valuable therapeutic option for difficult-to-treat Gram-negative infections. For instance, Hoellinger et al. [18] reported a 69% clinical cure rate in severe infections caused by non-fermenters, while Bavaro et al. [19] observed similar outcomes in a multicenter Italian series involving carbapenem-resistant *P. aeruginosa* and *A. baumannii*. More recent international cohorts [15,16,17] further corroborate these findings, showing comparable clinical outcomes in critically ill patients treated with either monotherapy or combination regimens. Although cefiderocol has a higher cost compared to conventional therapies, available pharmacoeconomic data suggest that its use may be justified in selected patients with MDR Gram-negative infections, particularly when it allows avoidance of more toxic regimens or reduces treatment complexity [20]. Our findings are also consistent with the previously reported in vitro activity of cefiderocol against MBL-producing Enterobacterales and *P. aeruginosa*, both of which were prevalent in our cohort. This was reflected in the observed MIC distributions and is supported by data from surveillance studies and clinical experience series [9,13,17].

Regarding less frequent infection sites, central nervous system infections were uncommon in our cohort and were limited to nosocomial ventriculitis. Clinical experience with cefiderocol in this setting remains scarce; however, its pharmacokinetic profile and the limited clinical evidence reports suggest that adequate drug exposure may be achievable in selected cases [20]. In this context, therapeutic drug monitoring data were available for one case of *S. maltophilia* ventriculitis and are currently under analysis.

Recent observational studies from the U.S. and Europe similarly reported sustained activity against these high-risk pathogens [22,23], supporting cefiderocol as a valuable therapeutic option when alternative agents are limited. Consistent with these findings, data from early-access programs have also demonstrated the effectiveness and safety of cefiderocol, including the Spanish PERSEUS study, which reported favorable outcomes in patients with severe MDR Gram-negative infections and few therapeutic alternatives [24].

Emergence of resistance during therapy was rare, occurring in only one case involving a *K. pneumoniae* ST147 strain harboring NDM, OXA-48, and ESBL enzymes. Although MLST was not systematically performed in this study, the ST147 isolate identified in our cohort is consistent with previously reported high-risk clones circulating in our area and in other European settings, particularly those associated with carbapenemase production such as OXA-48 and NDM [8,25]. This low incidence is consistent with multicenter cohorts reporting resistance rates of 2–5% [15,18]. Nevertheless, sporadic cases—particularly among high-risk clones—highlight the importance of ongoing microbiological surveillance and antimicrobial stewardship, especially during prolonged treatment or in settings with a high prevalence of MBL producers [8,23]. In Spain carbapenemase production in *P. aeruginosa* remains relatively uncommon, reported in approximately 2–3% of isolates in multicentre studies. However, when present, VIM-type enzymes account for the vast majority of cases (≈85–90%) [7]

In our cohort, 21.2% of patients received combination therapy, most commonly with aerosolized colistin, although most patients were treated with cefiderocol monotherapy. While some studies suggest that combination therapy may enhance microbiological clearance in carbapenem-resistant *A. baumannii* and MBL-producing Enterobacterales [26], observational multicenter studies have not demonstrated consistent survival benefits over monotherapy [19,21,22]. Consistent with these findings, we observed no significant differences in clinical outcomes between monotherapy and combination therapy groups, although larger prospective studies are needed to clarify this further. The role of combination therapy in preventing the emergence of resistance during cefiderocol treatment remains uncertain. While it has been proposed as a strategy to reduce selective pressure, current evidence does not consistently demonstrate a clear benefit over monotherapy [19,23].

Cefiderocol was generally well tolerated, with few adverse events necessitating treatment discontinuation. This aligns with safety data from randomized trials [10,11] and large observational series [14,16,22]. Across multicenter cohorts, serious adverse events appear to be uncommon, with treatment discontinuation rates ranging from 3% to 7% [16,21,27], supporting cefiderocol’s suitability for critically ill patients with complex comorbidities. Neurotoxic events, including myoclonus as observed in our cohort, have been only rarely reported during cefiderocol therapy, particularly among patients with renal dysfunction, consistent with the well-recognized neurotoxicity profile of β-lactams.

Our study adds meaningful evidence by providing detailed clinical, microbiological, and therapeutic data from a tertiary-care center managing highly resistant Gram-negative infections. However, several limitations must be acknowledged. The retrospective, single-center design may limit generalizability, and the absence of a comparator group restricts direct evaluation of cefiderocol’s effectiveness relative to other salvage therapies such as ceftazidime–avibactam or colistin-based regimens. Additionally, despite being larger than many previously published cohorts, our sample size remains insufficient for robust pathogen-specific or subgroup analyses. Microbiological characterization was based on routine diagnostic methods, as genomic analyses and multilocus sequence typing were not systematically performed, limiting detailed assessment of resistance mechanisms, circulating clones, and potential high-risk lineages. Furthermore, the microbiological distribution in our cohort, characterized by a predominance of non-fermenting Gram-negative bacilli and an underrepresentation of carbapenemase-producing Enterobacterales, particularly NDM-producing strains, may limit the external validity of our findings. This distribution likely reflects both local epidemiology and treatment selection practices, as cefiderocol use in our setting is influenced by clinical decision-making and the availability of alternative agents, particularly for Enterobacterales. In addition, the high proportion of critically ill patients with predominantly respiratory infections may have further contributed to this distribution. These findings should therefore be interpreted within this specific clinical context, which may differ from other settings with distinct epidemiological patterns or prescribing strategies in the context of the marked regional heterogeneity in carbapenemase epidemiology across Europe, as highlighted in recent genomic studies [7].

Overall, our findings support cefiderocol as an effective and generally safe option for treating severe MDR-GNB infections. Its sustained activity against MBL-producing pathogens, coupled with its favorable safety profile, positions it as a valuable component of the antimicrobial arsenal. Nonetheless, given limited therapeutic alternatives and the potential for emerging resistance, judicious use guided by antimicrobial stewardship remains essential. Continued monitoring of clinical outcomes and microbiological responses will be crucial to preserving its effectiveness. Future prospective multicenter studies should focus on defining the optimal role of monotherapy versus combination therapy, refining dosing strategies for critically ill patients, and strengthening resistance surveillance networks to enhance cefiderocol’s clinical utility.

## 4. Materials and Methods

This retrospective observational study was conducted at Bellvitge University Hospital in Barcelona, Spain, between November 2020 and October 2024, and included adult patients (≥18 years) who received at least 72 h of cefiderocol for documented MDR Gram-negative bacterial infections. Eligible patients were identified through a systematic review of electronic medical records. The study was carried out in accordance with institutional ethical standards.

Patients were eligible if they had received ≥72 h of cefiderocol and had a confirmed MDR Gram-negative infection with limited therapeutic alternatives. Exclusion criteria included missing essential clinical or microbiological data and death occurring within 48 h of obtaining the index culture. Multidrug-resistant (MDR) was defined as non-susceptibility to at least one agent in three or more antimicrobial categories [28].

Cefiderocol was administered as a standard 2 g intravenous infusion every 8 h over 3 h, with dosing adjustments according to renal function based on manufacturer recommendations. In cases of augmented renal clearance (creatinine clearance >120 mL/min), dosing was increased to 2 g every 6 h. Cefiderocol was used either as monotherapy or in combination with aerosolized colistin, aminoglycosides, or aztreonam-based regimens. De-escalation based on clinical progress or microbiological results was permitted. In polymicrobial infections, cefiderocol therapy was directed toward the multidrug-resistant Gram-negative pathogen considered clinically relevant by the treating physician.

Collected data included demographics, comorbidities (assessed using the Charlson Comorbidity Index), infection source, ICU admission, need for organ support (mechanical ventilation, cCRRT, or ECMO), and severity at infection onset (including septic shock). Infection site (e.g., respiratory, abdominal, bloodstream) and polymicrobial status were also recorded.

Microbiological identification was performed using standard laboratory techniques, including MALDI-TOF mass spectrometry (Bruker, Billerica, MA, USA). Antibiotic susceptibility testing employed automated systems (MicroScan WalkAway^®^, Beckman Coulter, Brea, CA, USA). Cefiderocol minimum inhibitory concentrations (MICs) were determined using gradient diffusion strips (Etest^®^, bioMérieux, Marcy-l’Étoile, France), according to the manufacturer’s instructions. Carbapenemase production was assessed using NG-Test^®^ CARBA-5 (NG Biotech, Guipry-Messac, France) and PCR assays targeting carbapenemase-encoding genes (Xpert^®^ Carba-R, Cepheid, Sunnyvale, CA, USA). Genomic analyses, including whole-genome sequencing and multilocus sequence typing, were not systematically performed as part of the study.

The primary outcome was clinical success, defined as survival and absence of clinical recurrence 30 days after initiating cefiderocol. Secondary outcomes included 30-day and 90-day all-cause mortality, 30-day and 90-day clinical recurrence, 90-day microbiological recurrence (re-isolation of the baseline pathogen), emergence of resistance to cefiderocol, and adverse events.

A descriptive analysis was conducted to characterize the clinical profile of enrolled patients. Means and standard deviations were used for normally distributed continuous variables, and medians with interquartile ranges for non-normally distributed variables. Comparisons between monotherapy and combination therapy groups employed the chi-square test for categorical variables and Student’s t test or Mann–Whitney U test for continuous variables, depending on data distribution.

## Figures and Tables

**Table 1:** Patient baseline characteristics at admission.

Patient Baseline Characteristics	*n* = 80
Age, Median (IQR)	64 (56–72)
Sex, Male (*n*, %)	65 (81.3%)
Charlson Comorbidity Index, Median (IQR)	3 (2–7)
Baseline Conditions	
Chronic liver disease	14 (17.5%)
Chronic renal disease	16 (20.0%)
Hypertension	41 (51.3%)
Diabetes mellitus	26 (32.5%)
Chronic Obstructive Pulmonary Diseases	6 (7.5%)
Chronic Cardiac Insufficiency	6 (7.5%)
Solid malignancy	15 (18.8%)
Hematological malignancy	3 (3.8%)
Immunosuppression	20 (25%)
Admission Background	
Major surgery in previous 30 days	17 (21.3%)
Hospital admission in previous 30 days	50 (62.5%)
Antibiotics during the previous 30 days	72 (90.0%)
One previous antibiotic	25 (31.3%)
Two or more previous antibiotics	46 (57.5%)
Reason for Hospital Admission	
Abdominal sepsis	14 (17.5%)
Urinary sepsis	11 (13.7%)
Acute pancreatitis	9 (11.2%)
Healthcare-related pneumonia	8 (10.0%)
Infectious endocarditis	4 (5.0%)
Acute subarachnoid hemorrhage	2 (2.5%)
Skin infection	3 (3.7%)
Solid organ transplantation	2 (2.5%)
Orthopedic elective surgery	2 (2.5%)
Esophageal perforation	1 (1.2%)
Acute lower limb ischemia	1 (1.2%)
Elective thoracic surgery	5 (6.2%)
Catheter-related bacteremia	1 (1.2%)
Tracheal fistula	1 (1.2%)
Prosthetic joint infection	5 (6.2%)
Abdominal surgical site infection	4 (5.0%)
Congestive cardiac insufficiency	3 (3.7%)
Acute subdural hematoma	2 (2.5%)
Necrotizing fasciitis	2 (2.5%)

Data are presented as *n* (%) unless otherwise indicated. IQR = Interquartile range.

**Table 2:** Clinical characteristics of the episode treated with cefiderocol.

Focus of Infection	*n* = 80
Respiratory	22 (27.5%)
Ventilator associated pneumonia	12 (15.0%)
Ventilator associated tracheobronchitis	10 (12.5%)
Abdominal	20 (25.0%)
Urinary	13 (16.2%)
Bone and joint infection	8 (10.0%)
**Skin and soft tissue infection**	6 (5.8%)
Catheter-related bloodstream infection	3 (3.7%)
Surgical site infection	5 (6.2%)
Central nervous system	3 (3.7%)
Severity Indicators	
ICU admission	36 (45.0%)
Septic shock	21 (26.3%)
Need for mechanical ventilation	27 (33.7%)
Renal replacement therapy	16 (20.0%)
**Use of extracorporeal membrane oxygenation**	5 (6.3%)
Bacteremia	16 (20.0%)
**Pitt bacteremia index, median (IQR)**	5 (3–7)

Data are presented as *n* (%) unless otherwise indicated. IQR = Interquartile range.

**Table 3:** Clinical endpoints and adverse events.

Primary and Secondary Endpoints	Total *n* = 80 (%)	Cefiderocol in Monotherapy *n* = 63 (78.10%)	Cefiderocol in Combination Therapy *n* = 17 (21.25%)	*p*-Value (CI 95%)
Clinical success *	54 (67.5%)	43 (68.3%)	11 (64.7%)	*p* = 0.78
30-day all-cause case-fatality rate	22 (27.5%)	17 (26.9%)	5 (29.2%)	*p* = 0.84
90-day all-cause case-fatality rate	29 (36.5%)	23 (36.5%)	6 (35.3%)	*p* = 0.92
30-day clinical recurrence	4 (5.0%)	3 (4.7%)	1 (5.8%)	*p* = 0.85
90-day clinical recurrence	2 (2.4%)	2 (3.1%)	0 (0%)	*p* = 0.45
90-day microbiological recurrence	1 (1.2%)	1 (1.6%)	0 (0%)	*p* = 0.60
Adverse events	4 (5.0%)			
Rash	1 (1.2%)			
Dizziness	1 (1.2%)			
Myoclonic movements	1 (1.2%)			
Thrombocytopenia	1 (1.2%)			

* Clinical success defined as survival and absence of clinical recurrence at 30 days following cefiderocol initiation. Clinical success rate by focus: respiratory 68.2% (VAP 41.7%), abdominal 40%, urinary 53.8%, bone and joint infection 50%, SSTI 33.3%, CRBSI 66.7%, and CNS 33.3%.

## Data Availability

The datasets generated and/or analyzed during the current study are not publicly available to preserve the individual privacy of the participants, but they are available from the corresponding author.

## Supplementary Materials

The following supporting information can be downloaded at: https://www.mdpi.com/article/10.3390/antibiotics15040416/s1, Table S1. Distribution of microorganisms and resistance mechanisms in monomicrobial infections; Table S2. Distribution of microorganisms and resistance mechanisms in polymicrobial infections; Table S3. Cefiderocol MIC50 values and MIC ranges by pathogen; Table S4. Combination therapy with cefiderocol according to antibiotic and infection source; Table S5. Clinical outcomes by infection type and causative pathogen; Table S6. Characteristics of patients according to treatment outcome.

## References

[1] Macesic N., Uhlemann A.-C., Peleg A.Y. (2025). Multidrug-resistant Gram-negative bacterial infections. Lancet.

[2] World Health Organization, European Centre for Disease Prevention and Control (2022). Antimicrobial Resistance Surveillance in Europe 2022, 2020 Data.

[3] European Centre for Disease Prevention and Control (ECDC) (2024). Antimicrobial Resistance in the EU/EEA (EARS-Net) Annual Epidemiological Report 2024.

[4] Grundmann H., Glasner C., Albiger B., Aanensen D.M., Tomlinson C.T., Andrasević A.T., Carmeli Y., Friedrich A.W., Giske C.G., Glupczynski Y. (2017). Occurrence of carbapenemase-producing Enterobacteriaceae in Europe (EuSCAPE). Lancet Infect. Dis..

[5] Logan L.K., Weinstein R.A. (2017). The epidemiology of carbapenem-resistant Enterobacteriaceae: The Impact and Evolution of aGlobal Menace. Clin. Infect. Dis..

[6] van Duin D., Doi Y. (2017). The global epidemiology of carbapenemase-producing Enterobacteriaceae. Virulence.

[7] Gracia-Ahufinger I., López-González L., Vasallo F.J., Galar A., Siller M., Pitart C., Bloise I., Torrecillas M., Gijón-Cordero D., Viñado B. (2023). The CARBA-MAP study: National mapping of carbapenemases in Spain (2014–2018). Front. Microbiol..

[8] Budia-Silva M., Kostyanev T., Ayala-Montaño S., Bravo-Ferrer Acosta J., Garcia-Castillo M., Cantón R., Goossens H., Rodriguez-Baño J., Grundmann H., Reuter S. (2024). International and regional spread of carbapenem-resistant *Klebsiella pneumoniae* in Europe. Nat. Commun..

[9] McCreary E.K., Heil E.L., Tamma P.D. (2021). New Perspectives on Antimicrobial Agents: Cefiderocol. Antimicrob. Agents Chemother..

[10] Bassetti M., Echols R., Matsunaga Y., Ariyasu M., Doi Y., Ferrer R., Ariyoshi T., Naas T., Niki Y., Paterson D.L. (2021). Efficacy and safety of cefiderocol (CREDIBLE-CR). Lancet Infect. Dis..

[11] Wunderink R.G., Matsunaga Y., Ariyasu M., Clevenbergh P., Echols R., Kaye K.S., Belley A., Menon A., Pogue J.M., Shorr A.F. (2021). APEKS-NP trial of cefiderocol vs meropenem. Lancet Infect. Dis..

[12] Portsmouth S., van Veenhuyzen D., Echols R., Machida M., Ferreira J.C.A., Ariyasu M., Tenke P., Nagata T.D. (2018). Cefiderocol versus imipenem-cilastatin for the treatment of complicated urinary tract infections caused by Gram-negative uropathogens: A phase 2, randomised, double-blind, non-inferiority trial. Lancet Infect. Dis..

[13] Timsit J.F., Paul M., Shields R.K., Echols R., Baba T., Yamano Y., Portsmouth S. (2022). Cefiderocol for the Treatment of Infections Due to Metallo-B-lactamase-Producing Pathogens in the CREDIBLE-CR and APEKS-NP Phase 3 Randomized Studies. Clin. Infect. Dis..

[14] Hsueh S.C., Chao C.M., Wang C.Y., Lai C.C., Chen C.H. (2021). Systematic review and meta-analysis of cefiderocol. J. Glob. Antimicrob. Resist..

[15] Piccica M., Spinicci M., Botta A., Bartalesi F., Mantella A., Leonildi A., Faragona A., Parrella R., Giani T., Bartolini A. (2023). Italian real-life cefiderocol experience. J. Antimicrob. Chemother..

[16] Babidhan R., Lewis A., Atkins C., Jozefczyk N.J., Nemecek B.D., Montepara C.A., Gionfriddo M.R., Zimmerman D.E., Covvey J.R., Guarascio A.J. (2022). Safety and efficacy of cefiderocol for off-label treatment indications: A systematic review. Pharmacotherapy.

[17] Fendian Á.M., Albanell-Fernández M., Tuset M., Pitart C., Castro P., Soy D., Bodro M., Soriano A., Del Río A., Martínez J.A. (2023). Real-Life Data on the Effectiveness and Safety of Cefiderocol in Severely Infected Patients: A Case Series. Infect. Dis. Ther..

[18] Hoellinger B., Simand C., Jeannot K., Garijo C., Cristinar M., Reisz F., Danion F., Ursenbach A., Lefebvre N., Boyer P. (2023). Real-world clinical outcome of cefiderocol for treatment of multidrug-resistant non-fermenting, gram negative bacilli infections: A case series. Clin. Microbiol. Infect..

[19] Bavaro D.F., Belati A., Diella L., Stufano M., Romanelli F., Scalone L., Stolfa S., Ronga L., Maurmo L., Dell’Aera M. (2021). Cefiderocol-Based Combination Therapy for “Difficult-to-Treat” Gram-Negative Severe Infections: Real-Life Case Series and Future Perspectives. Antibiotics.

[20] Bilal M., El Tabei L., Büsker S., Krauss C., Fuhr U., Taubert M. (2021). Pharmacokinetic/pharmacodynamic considerations and cost-effectiveness of cefiderocol in the treatment of multidrug-resistant Gram-negative infection. Clin. Pharmacokinet..

[21] Meschiari M., Volpi S., Faltoni M., Dolci G., Orlando G., Franceschini E., Menozzi M., Sarti M., Del Fabro G., Fumarola B. (2021). Real-life experience with compassionate use of cefiderocol for difficult-to-treat resistant *Pseudomonas aeruginosa* (DTR-P) infections. JAC Antimicrob. Resist..

[22] Clancy C.J., Cornely O.A., Marcella S.W., Nguyen S.T., Gozalo L., Cai B. (2024). Effectiveness and Safety of Cefiderocol in Clinical Practice for Treatment of Patients with Gram-Negative Bacterial Infections: US Interim Results of the PROVE Study. Infect. Drug Resist..

[23] Soueges S., Faure E., Parize P., Lanternier-Dessap F., Lecuyer H., Huynh A., Martin-Blondel G., Gaborit B., Blot M., Magallon A. (2025). G2I (Groupe Immunodepression et Infections) network. Real-world multicentre study of cefiderocol treatment of immunocompromised patients with infections caused by multidrug-resistant Gram. Negative bacteria: CEFI-ID study. J. Infect..

[24] Torre-Cisneros J., Almirante B., Martos C.F., Rascado P., Lletí M.S., Sánchez-García M., Soriano A., Soriano-Cuesta M.C., Gonzalez Calvo A.J., Karas A. (2025). Effectiveness and safety of cefiderocol treatment in patients with Gram-negative bacterial infections in Spain in the early access programme: Results of the PERSEUS study. Eur. J. Clin. Microbiol. Infect. Dis..

[25] Shaw E., Rombauts A., Tubau F., Padullés A., Càmara J., Lozano T., Cobo-Sacristán S., Sabe N., Grau I., Rigo-Bonnin R. (2018). Clinical outcomes after combination treatment with ceftazidime/avibactam and aztreonam for NDM-1/OXA-48/CTX-M-15-producing *Klebsiella pneumoniae* infection. J. Antimicrob. Chemother..

[26] Gatti M., Bartoletti M., Cojutti P.G., Gaibani P., Conti M., Giannella M., Viale P., Pea F. (2021). A descriptive case series of pharmacokinetic/pharmacodynamic target attainment and microbiological outcome in critically ill patients with documented severe extensively drug-resistant *Acinetobacter baumannii* bloodstream infection and/or ventilator-associated pneumonia treated with cefiderocol. J. Glob. Antimicrob. Resist..

[27] Chou A., Ramsey D., Amenta E., Trautner B.W. (2022). Real-world experience with cefiderocol therapy for *Pseudomonas aeruginosa* and other multidrug resistant gram-negative infections within the Veterans Health Administration, 2019–2022. Antimicrob. Steward. Healthc. Epidemiol..

[28] Magiorakos A.P., Srinivasan A., Carey R.B., Carmeli Y., Falagas M.E., Giske C.G., Harbarth S., Hindler J.F., Kahlmeter G., Olsson-Liljequist B. (2012). Multidrug-resistant, extensively drug-resistant and pandrug-resistant bacteria: An international expert proposal for interim standard definitions for acquired resistance. Clin. Microbiol. Infect..

