# Supplementary material for: Cefiderocol Targeted Treatment for Multidrug-Resistant Gram-Negative Infections: An Observational Cohort Study
Source: Antibiotics (Basel). 2026 Apr 20;15(4):416. doi: 10.3390/antibiotics15040416 (PMC13113607; doi:10.3390/antibiotics15040416)
Supplement: Supplementary file 1 [file antibiotics-15-00416-s001.zip › antibiotics-4246133-supplementary.pdf]

Supplementary Tables

Cefiderocol targeted treatment for multidrug-resistant Gram-negative infections: An Observational Cohort Study

Table S1. Distribution of microorganisms and resistance mechanisms in monomicrobial infections (n = 58 isolates)

**Microorganism**	**Resistance mechanism / phenotype**	**n**
Pseudomonas aeruginosa	VIM-type metallo-β-lactamase	9
	Extremely drug-resistant (XDR)	9
	Multidrug-resistant (MDR)	6
Stenotrophomonas maltophilia	Intrinsic multidrug resistance	24
Klebsiella pneumoniae	OXA-48 + VIM β-lactamases	3
	NDM metallo-β-lactamase	1
	KPC carbapenemase	1
Other Enterobacterales	OXA-48 carbapenemase	2
Burkholderia cenocepacia	Intrinsic multidrug resistance	2
Acinetobacter baumannii	Wild type phenotipe	1

*Data are presented as number of isolates (n). MDR: multidrug-resistant; XDR: extremely drug-resistant; ESBL: extended-spectrum β-lactamase; MBL: metallo-β-lactamase; VIM: Verona integron-encoded metallo-β-lactamase; NDM: New Delhi metallo-β-lactamase; OXA-48: oxacillinase-48; KPC: Klebsiella pneumoniae carbapenemase. Resistance mechanisms were inferred from phenotypic susceptibility testing and targeted molecular assays routinely used in clinical practice. Whole-genome sequencing was not performed.

Table S2. Distribution of microorganisms and resistance mechanisms in polymicrobial infections (n = 45 isolates)

Microorganism	Resistance mechanism / phenotype	n
Pseudomonas aeruginosa	VIM-type metallo-β-lactamase	7
	Extremely drug-resistant (XDR)	4
Stenotrophomonas maltophilia	Intrinsic multidrug resistance	7
Klebsiella pneumoniae	ESBL + OXA-48 + VIM	5
	ESBL + NDM	3
	ESBL (no carbapenemase)	5
Escherichia coli	ESBL	4
	ESBL + NDM	1
Enterobacter cloacae	ESBL + VIM	4

Microorganism	Resistance mechanism / phenotype	n
Klebsiella oxytoca	ESBL + VIM	3
Serratia marcescens	Wild type phenotipe	1

* A total of 45 isolates were recovered from 22 polymicrobial infections with the following combinations: *S. maltophilia* + *K. pneumoniae* ESBL-OXA48-VIM (n = 3); *S. maltophilia* + *S. marcescens* (n = 1); *S. maltophilia* + *E. coli* ESBL (n = 3); *P. aeruginosa* VIM + *K. oxytoca* ESBL-VIM (n = 1); *P. aeruginosa* VIM + *K. pneumoniae* ESBL-NDM (n = 3); *P. aeruginosa* VIM + *E. cloacae* ESBL-VIM (n = 2); *P. aeruginosa* VIM + *E. coli* ESBL-NDM (n = 1); *K. pneumoniae* ESBL-OXA48-VIM + *E. coli* ESBL (n = 1); *K. pneumoniae* ESBL-NDM + *K. pneumoniae* ESBL (n = 1); *E. cloacae* ESBL-VIM + *K. pneumoniae* ESBL (n = 1); *P. aeruginosa* XDR + *K. oxytoca* ESBL-VIM (n = 2); *P. aeruginosa* XDR + *K. pneumoniae* ESBL (n = 2); *E. cloacae* ESBL-VIM + *K. pneumoniae* ESBL + *K. pneumoniae* ESBL-OXA48-VIM (n = 1).

**Data are presented as number of isolates (n). Each microorganism in polymicrobial infections was counted individually. MDR: multidrug-resistant; XDR: extremely drug-resistant; ESBL: extended-spectrum β-lactamase; MBL: metallo-β-lactamase; VIM: Verona integron-encoded metallo-β-lactamase; NDM: New Delhi metallo-β-lactamase; OXA-48: oxacillinase-48; KPC: Klebsiella pneumoniae carbapenemase. Resistance mechanisms were inferred from phenotypic susceptibility testing and targeted molecular assays routinely used in clinical practice. Whole-genome sequencing was not performed.

Table S3. Cefiderocol MIC50 values and MIC ranges by pathogen

Pathogen	n (available) / n (total)	MIC50 (mg/L)	Range (mg/L)
Enterobacterales	16/18	0.25	0.016–1
S. maltophilia	14/24	0.047	0.016–0.25
P. aeruginosa	17/35	0.38	0.023–2

Pathogen	n (available) / n (total)	MIC50 (mg/L)	Range (mg/L)
B. cenocepacea	2/2	1	1 – 2
A. baumannii	1/1	0.5	0.5 – 2

*Cefiderocol minimum inhibitory concentrations (MICs) were determined by gradient diffusion (Etest), as described in the Methods section. MIC data were available for 50 of the 80 isolates included in the study. MIC50 values and ranges are reported as descriptive measures.

Table S4. Combination therapy with cefiderocol according to antibiotic and infection source (n = 17)

Combination therapy	Antibiotic	Infection source	n
Cefiderocol + Amikacin	Amikacin (intravenous)	Bloodstream	2
		Urinary	1
		Respiratory	1
Cefiderocol + intravenous colistin	Colistin (intravenous)	Intra-abdominal	2
Cefiderocol + aerosolized colistin	Colistin (aerosolized)	Respiratory	11

*Data are presented as number of patients (n). A total of 17 out of 80 patients (21.2%) received combination therapy. Combination therapy was defined as the concomitant administration of cefiderocol with another active antimicrobial agent for at least 48 hours.

Table S5. Clinical outcomes by infection type and causative pathogen

Category	Clinical success, n (%)	30-day all-cause case-fatality rate	90-day all-cause case-fatality rate n (%)	30-day Clinical recurrence n (%)	90-day Clinical recurrence n (%)	90-day Microbiological recurrence (90 days), n (%)
**Infection type**						
Monomicrobial	44 (75.9%)	17 (29.3%)	16 (27.6%)	1 (1.7%)	1 (1.7%)	1 (1.7%)
Polymicrobial	10 (45.5%)	5 (22.7%)	13 (59.1%)	3 (13.6%)	1 (4.5%)	0 -
**Main causative Pathogen***						
Enterobacterales	10/18 (55.5%)	6/18 (33.3%)	7/18 (38.9%)	2/18 (11.1%)	1/18 (5.6%)	1/18 (5.6%)
Stenotrophomonas maltophilia	18/24 (75.0%)	4/24 (16.7%)	5/24 (20.8%)	1/24 (4.2%)	1/24 (4.2%)	0 (0.0%)
Pseudomonas aeruginosa	23/35 (65.0%)	9/35 (25.7%)	16/35 (45.7%)	2/35 (5.7%)	0 (0.0%)	0 (0.0%)
Burkholderia cenocepacia	2/2 (100%)	0 (0.0%)	0 (0.0%)	0 (0.0%)	0 (0.0%)	0 (0.0%)
Acinetobacter baumannii	1/1 (100%)	0 (0.0%)	0 (0.0%)	0 (0.0%)	0 (0.0%)	0 (0.0%)

*Outcomes are reported per patient. In polymicrobial infections, outcomes may be attributed to more than one pathogen; therefore, pathogen-specific outcomes do not sum to overall totals. Clinical success was defined as survival and absence of clinical recurrence at 30 days after cefiderocol initiation. Percentages should be interpreted with caution due to limited subgroup sizes

Table S6. Characteristics of patients according to treatment outcome

	Overall (n=80)	Clinical success (n=54; 67.5%)	Treatment failure (n=26;32.5%)
**Demographics**			
Age, years (median, IQR)	64 (56–72)	63 (56 – 76)	68 (58–78)
**Comorbidity**			
Charlson index (median, IQR)	3 (2–7)	3 (2-7)	3 (2–8)
**Clinical severity**			
Septic shock, n (%)	21 (26.3%)	5 (9.3%)	16 (61.5%)
ICU admission, n (%)	36 (45.0%)	22 (40.7%)	14 (53.8%)
**Microbiology**			
P. aeruginosa VIM-producing, n (%)	16 (15.5%)	8 (14.8%)	8 (30.8%)
XDR *P. aeruginosa*, n (%)	13 (12.6%)	7 (12.9%)	6 (23.1%)
Enterobacterales, n (%)	34 (33.0%)	22 (40.7%)	12 (46.1%)
Polymicrobial infection, n (%)	22 (27.5%)	6 (11.1%)	16 (61.5%)
**Infection source**			
Intra-abdominal infection, n (%)	20 (25.0%)	8 (14.8%)	12 (46.2%)

	Overall (n=80)	Clinical success (n=54; 67.5%)	Treatment failure (n=26;32.5%)
Respiratory infection	22 (27.5%)	10 (18.5%)	12 (46.2%)
**Treatment**			
Combination therapy, n (%)	17 (21.2%)	7 (13.0%)	10 (38.5%)

*Data are presented as median (interquartile range) or number of patients (%). Percentages are calculated within each group. Clinical success and treatment failure groups were defined according to the primary outcome. No formal statistical comparisons were performed due to the limited sample size.
